# Comparison of the prophylactic effect of dexamethasone and dexmedetomidine and their combination in reducing postoperative nausea and vomiting in patients undergoing laparoscopic cholecystectomy

**DOI:** 10.25122/jml-2020-0030

**Published:** 2021

**Authors:** Siamak Rekei, Amir Reza Naeimi, Behnam Mahmodiyeh, Roya Golmoradi, Alireza Kamali

**Affiliations:** 1. Department of Surgery, Arak University of Medical Sciences, Arak, Iran; 2. Department of Anesthesiology and Critical Care, Arak University of Medical Sciences, Arak, Iran

**Keywords:** dexmedetomidine, dexamethasone, vomiting, nausea, laparoscopy

## Abstract

Nausea and vomiting are some of the most common complaints of patients after any anesthesia, which is often associated with postoperative pain. The double-blind clinical trial study aimed to compare the prophylactic effect of dexamethasone and dexmedetomidine and their combination in reducing postoperative nausea and vomiting in patients undergoing laparoscopic cholecystectomy. One hundred sixty-two patients undergoing laparoscopic cholecystectomy were enrolled in the study. In the first group of patients, 25 mg of dexmedetomidine were administered slowly. In comparison, the patients in the second group received dexamethasone (4 ml/2 mg) with 0.1 mg/kg of normal saline solution. The third group received a combination of dexmedetomidine and dexamethasone. Hemodynamic changes were recorded during surgery and after surgery, and the patients were admitted to recovery. Nausea and vomiting scores were recorded 2 and 4 hours after surgery. Blood pressure and heart rate were lower in the dexmedetomidine group at all times (P<0.05). Two hours after surgery, the dexamethasone and dexmedetomidine combination group had less vomiting (P=0.012). The incidence of nausea 2 and 4 hours after surgery was lower in the dexamethasone and dexmedetomidine combination group (P<0.05). Blood pressure and heart rate were lower in the dexmedetomidine group at all times. The dexmedetomidine and dexamethasone combination decreased postoperative nausea and vomiting in patients. Therefore, we recommend using a dexmedetomidine and dexamethasone combination for reducing postoperative nausea and vomiting.

## INTRODUCTION

Nausea and vomiting are some of the most common complaints of patients after any anesthesia, which is often associated with postoperative pain [1]. Postoperative nausea and vomiting may occur up to 24 hours after surgery, occurring in 20–30% of patients [2]. This may be due to the effect of anesthetics on the vomiting control center in the medulla oblongata or because of a decrease in intraoperative hypoxia which subsequently causes nausea and vomiting. In Germany, every year, 8 million surgeries are performed, about 2.4 million people suffer from postoperative nausea and vomiting (PONV). Postoperative nausea and vomiting can cause complications such as airway obstruction, aspiration pneumonia, and surgical wound opening [3, 4]. Postoperative vomiting causes dehydration, electrolyte abnormalities, hypertension, suture stretching, increased bleeding from skin flaps, and delayed discharge. This complication can increase the risk of pulmonary aspiration if the airway reflexes are reduced due to the residual effects of anesthetic drugs [2]. To date, several drugs, including dopamine and serotonin receptor antagonists, corticosteroids, antihistamines, sedatives and anticholinergics, have been used to treat this disorder [5–7]. The most common drugs to relieve nausea and vomiting are metoclopramide and droperidol. Due to complications such as fatigue and restlessness, lack of awareness of time and place, extrapyramidal symptoms, cardiovascular complications, hypotension, orthostatic hypotension, drowsiness, akathisia, elevated liver enzymes, and agranulocytosis, these agents have been limited in some cases [8]. Nowadays, new therapies have replaced the above-mentioned drugs, such as drug therapy and complementary therapies, to be used as a stand-alone treatment or in combination with standard treatments [6]. Dexmedetomidine, a potent alpha-2-adrenergic receptor agonist, has been widely used due to its anxiolytic, sedative, analgesic, sympatholytic, and hemodynamic regulation properties [9]. During surgery, dexmedetomidine can reduce the incidence of restlessness and provide an acceptable recovery. It also reduces postoperative pain without hemodynamic complications. It can prevent postoperative nausea and vomiting [9], and numerous studies have shown the anti-nausea and vomiting effects of low-dose dexmedetomidine [10–12]. Dexamethasone is a corticosteroid drug that reduces inflammation and weakens the immune system [13, 14]. Dexamethasone is a cheap and available drug that is used to control postoperative nausea and vomiting [15, 16]. During the last two decades, the anti-nausea and vomiting effects of dexamethasone have been shown in patients undergoing chemotherapy. Based on these findings, anesthesiologists' intention to use dexamethasone to reduce the incidence and severity of postoperative nausea and vomiting has been investigated [17, 18]. The results indicate a positive role of dexamethasone in reducing nausea and vomiting in different population groups. Although most studies on postoperative nausea and vomiting have been performed under general anesthesia, however, in other population groups, dexamethasone has been shown to reduce the incidence of nausea and vomiting [19–21]. Dexamethasone is an effective drug for nausea and vomiting, whose exact mechanism of preventing nausea and vomiting is still unknown. However, dexamethasone probably decreased postoperative nausea and vomiting by inhibiting prostaglandins [22, 23].

Since there is no study comparing these effective drugs in preventing nausea and vomiting so far, dexmedetomidine has other useful properties besides decreasing nausea and vomiting and can be a good alternative to other drugs. This study aimed to compare the prophylactic effect of dexamethasone and dexmedetomidine and their combination in reducing postoperative nausea and vomiting in patients undergoing laparoscopic cholecystitis.

## MATERIAL AND METHODS

This was a double-blind clinical trial. We included all laparoscopic candidates who gave informed consent, ASA Class I and II patients, those with no history of psychotic illnesses, no Parkinson's disease, motion disorder, or history of chemotherapy, patients under general anesthesia, patients aged 18–60 years, and a maximum surgical duration of 150 minutes. Exclusion criteria included patients who have not given their informed consent to be included in the study, patients with Parkinson's disease, psychotic illnesses, or those who had a history of chemotherapy. All patients were enrolled in the study after obtaining informed consent and entered the operating room after anesthesia confirmation. For these patients, oxygen saturation (SPO2), pulse rate (PR), blood pressure (BP), non-invasive blood pressure (NIBP), and body temperature were recorded. All patients were given 3–5 ml/kg of crystalloid fluid as compensatory volume expansion and the patients underwent general anesthesia. All patients received 2 mg/kg of fentanyl, 0.3–0.5 μg/kg of midazolam, 0.5–0.7 µg/kg of atracurium, and 2–3 mg/kg of propofol and underwent general anesthesia.

After patient intubation, endotracheal fixation, and hemodynamic stabilization, and prior to surgical excision, 25 μg of dexmedetomi-dine (Hospira, United States of America) were administered to patients in group I. Patients in group II were administered dexamethasone (Alborz Darou, Iran) (4 ml/2 mg) with 0.1 mg/kg solution of normal saline and a total volume of 20 ml. The third group received a mix of dexmedetomidine and dexamethasone.

After the surgery was completed and patients were admitted to recovery, questionnaires that included questions about their nausea and vomiting score (Table 1) and their hemodynamics were distributed to all patients. Finally, the data obtained from the questionnaires were analyzed using the Statistical Package for the Social Sciences (SPSS) software, version 23.

**Table 1:** Vomiting score.

Vomiting score	Description
**0**	No vomiting or any similar symptoms
**1**	Mild yaw that is manageable
**2**	Moderate yawning and vomiting 1 to 2 times, which is manageable
**3**	Frequent yawning and vomiting, which is difficult to control
**4**	Uncontrollable, recurrent vomiting

To blind the drug product, the anonymous syringe was drawn by an anesthesiologist and was given to the surgical resident, who then gave the injection to patients. Neither surgery residents nor patients knew in which groups they were assigned.

## RESULTS

This study was a double-blind clinical trial that included 164 candidates for laparoscopic surgery in Valiasr Hospital, Arak. The patients were randomly divided into three groups. The minimum age was 33 years, and the maximum age was 60 years. The mean age was 49.31±7.81 years. There was no statistically significant difference in the duration of surgery in the three groups (P=0.679).

According to Table 2, there was no significant difference in age between the three groups (P=0.783).

**Table 2:** Comparison of mean and standard deviation of age in dexamethasone, dexmedetomidine and drug combination groups.

Group Quantitative variable	Drug combination Mean±SD	Dexmedetomidine Mean±SD	Dexamethasone Mean±SD	P-value
**Age**	49.01±7.02	49.00±7.80	49.92±8.65	0.783

As shown in Table 3, there was no significant difference in gender in the four groups (P=0.358). Laparoscopic surgery is more common in women than in men, as known from previous studies.

**Table 3:** Comparison of frequency and sex in dexamethasone, dexmedetomidine and drug combination groups.

Group Gender	Drug combination (%)n	Dexmedetomidine (%)n	Dexamethasone (%)n	P-value
**Female**	43 (79.62)	48 (88.88)	47 (87.03)	0.358
**Male**	11 (20.37)	6 (11.11)	7 (12.96)	

**Figure 1:**
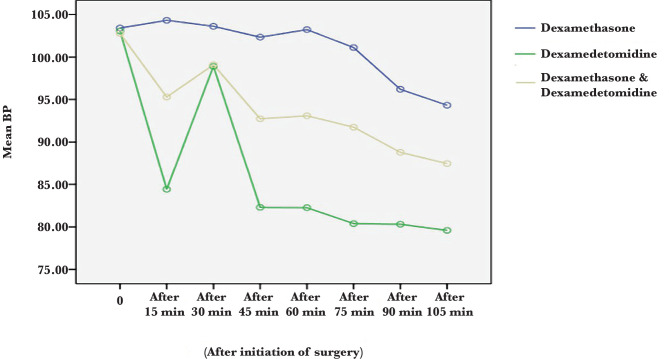
Comparison of mean blood pressure in dexamethasone, dexmedetomidine and drug combination groups.

As shown in Table 4 and Figure 1, there was a statistically significant difference in blood pressure at all times except at baseline in the three groups (P<0.05). Blood pressure was lower in the dexmedetomidine group at all times. Subsequently, the drug combination group had lower blood pressure than the dexamethasone group.

**Table 4:** Comparison of mean and standard deviation of blood pressure in dexamethasone, dexmedetomidine and drug combination groups.

Group BP	Drug combination Mean±SD	Dexmedetomidine Mean±SD	Dexamethasone Mean±SD	P-value
**Initiation**	84.25±7.46	85.98±7.17	86.53±7.62	0.254
**15 minutes after surgery initiation**	79.66±6.90	76.22±4.63	82.25±7.02	0.0001
**30 minutes after surgery initiation**	79.11±7.94	73.25±4.80	83.66±6.11	0.0001
**45 minutes after surgery initiation**	77.00±7.95	70.74±3.62	82.29±6.11	0.0001
**60 minutes after surgery initiation**	77.38±8.89	69.50±4.58	83.55±5.34	0.0001
**75 minutes after surgery initiation**	76.75±8.19	70.35±5.37	82.61±4.91	0.0001
**90 minutes after surgery initiation**	77.14±7.35	70.75±5.10	82.68±3.98	0.0001
**105 minutes after surgery initiation**	78.57±8.77	71.16±5.56	85.14±3.72	0.0001

**Figure 2:**
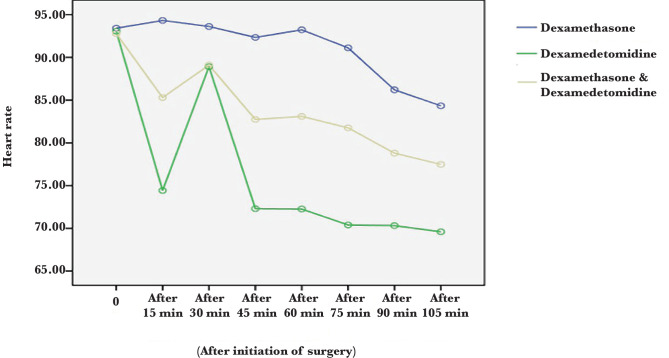
Comparison of mean heart rate in dexamethasone, dexmedetomidine and drug combination groups.

**Figure 3:**
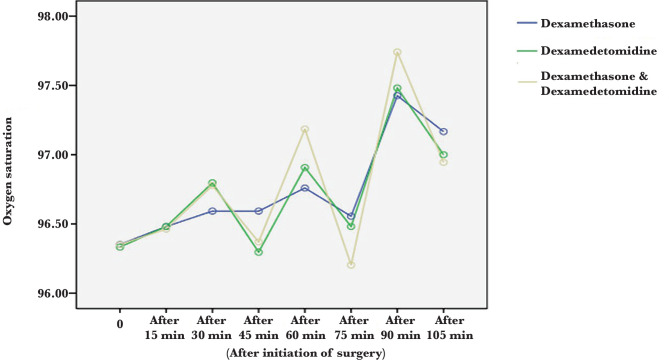
Comparison of mean oxygen saturation percentage in dexamethasone, dexmedetomidine and drug combination groups.

Heart rate at all times was statistically significant between the three groups except at baseline (P<0.05). The lowest heart rate was seen in the dexmedetomidine group. After dexmedetomidine, the drug combination group had a lower heart rate than the dexamethasone group (Table 5, Figure 2) However, as seen in Table 6 and Figure 3, there was no statistically significant difference in the percentage of oxygen saturation at all times (P>0.05).

**Table 5:** Comparison of mean and standard deviation of heart rate in dexamethasone, dexmedetomidine and drug combination groups.

Group Heart rate	Drug combination Mean±SD	Dexmedetomidine Mean±SD	Dexamethasone Mean±SD	P-value
**Initiation**	92.77±5.58	93.05±3.30	93.40±5.44	0.799
**15 minutes after surgery initiation**	85.29±11.85	74.42±9.04	94.31±4.29	0.0001
**30 minutes after surgery initiation**	89.09±5.52	88.90±6.15	93.61±4.52	0.0001
**45 minutes after surgery initiation**	82.74±12.20	72.29±9.33	92.33±3.81	0.003
**60 minutes after surgery initiation**	83.09±12.64	72.25±9.45	93.22±3.55	0.014
**75 minutes after surgery initiation**	81.75±11.78	70.38±8.18	91.11±3.79	0.0001
**90 minutes after surgery initiation**	78.79±9.75	70.31±7.46	86.20±3.95	0.0001
**105 minutes after surgery initiation**	77.48±9.68	69.59±8.94	84.33±3.66	0.0001

**Table 6:** Comparison of mean and standard deviation of oxygen saturation percentage in dexamethasone, dexmedetomidine and drug combination groups.

Group Oxygen saturation	Dexamethasone Mean±SD	Dexmedetomidine Mean±SD	Drug combination Mean±SD	P-value
**Initiation**	96.35±0.587	96.33±0.614	96.35±0.587	0.983
**15 minutes after surgery initiation**	96.48±0.74	96.48±0.745	96.46±0.745	0.989
**30 minutes after surgery initiation**	98.59±0.714	96.79±0.6555	96.77±1.609	0.560
**45 minutes after surgery initiation**	96.59±0.789	96.29±0.964	96.35±0.827	0.169
**60 minutes after surgery initiation**	96.75±0.547	96.90±0.783	97.18±1.93	0.200
**75 minutes after surgery initiation**	96.55±0.603	96.48±0.665	96.20±1.64	0.835
**90 minutes after surgery initiation**	97.42±0.716	97.48±1.023	97.74±0.974	0.210
**105 minutes after surgery initiation**	97.16±0636	97.00±0.582	96.94±0.563	0.165

There was no statistically significant difference regarding vomiting in recovery and 4 hours after surgery in the three groups (P>0.05) (Table 7). A significant difference was observed 2 hours after surgery (p=0.012). In the combination group of dexamethasone and dexmedetomidine, vomiting was less frequently reported compared to the other groups.

**Table 7:** Comparison of mean and standard deviation of vomiting in dexamethasone, dexmedetomidine and drug combination groups.

Vomiting Group	Dexamethasone Mean±SD	Dexmedetomidine Mean±SD	Drug combination Mean±SD	P-value
**Recovery**	0.562±0.203	0.419±0.222	0.264±0.074	0.156
**2 hour after surgery termination**	0.406±0.203	0.460±0.296	0.264±0.074	0.012
**4 hour after surgery termination**	0.406±0.203	0.358±0.148	0.264±0.074	0.155

The incidence of nausea during recovery was not significantly different between the three groups (P>0.05). The incidence of nausea at 2 and 4 hours after surgery was statistically significant between the three groups (P<0.05), being lower in the combination group (dexamethasone and dexmedetomidine) compared to the other two groups (Table 8).

**Table 8:** Comparison of frequency and incidence of nausea in dexamethasone, dexmedetomidine and drug combination groups.

Nausea Group		Dexamethasone Mean±SD	Dexmedetomidine Mean±SD	Drug combination Mean±SD	P-value
**Recovery**	Yes	11	14	12	0.783
	No	43	40	42	
**2 hours after surgery termination**	Yes	15	20	8	0.032
	No	39	34	46	
**4 hours after surgery termination**	Yes	11	20	8	0.019
	No	43	34	46	

## DISCUSSION

The purpose of this study was to compare the prophylactic effect of dexamethasone and dexmedetomidine and the combination of both in reducing postoperative nausea and vomiting in patients undergoing laparoscopic cholecystitis. The number of women in this study was higher, which is in line with the high prevalence of laparoscopic surgery in women.

We found that blood pressure was lower in the dexmedetomidine group at all times. In the next step, the combination of the two drugs showed lower blood pressure than the dexamethasone group. The lowest heart rate was seen in the dexmedetomidine group. After dexmedetomidine, the combination group had a lower heart rate compared to the dexamethasone group. Two hours after surgery, the vomiting was less frequent in the combination group. The incidence of nausea at 2 and 4 hours after surgery was lower in the dexamethasone and dexmedetomidine combination group than in the other two groups. Dexmedetomidine is one of the potent alpha-2-adrenergic receptor agonists that have been widely used due to its anxiolytic, sedative, analgesic, sympatholytic and hemodynamic regulation properties [9].

During surgery, dexmedetomidine can reduce the incidence of restlessness and provide an acceptable recovery. It also reduces postoperative pain without hemodynamic complications so that it can prevent postoperative nausea and vomiting [9]. The anti-nausea and vomiting effect of low-dose dexmedetomidine in reducing postoperative nausea and vomiting has been proven in numerous studies [10–12]. In our study, the combination between dexmedetomidine and dexamethasone decreased postoperative nausea and vomiting and decreased more significantly blood pressure and heart rate compared to the agents given alone.

In 2017, Shenhui *et al*. conducted a review to evaluate the role of dexmedetomidine in preventing nausea and vomiting in general anesthesia. The authors suggested that it could be used to reduce nausea and vomiting if the dexmedetomidine side effects could be reduced [24] and their results were in line with our study. Kleif *et al*. also conducted a study aimed to define the effect of preoperative dexamethasone on postoperative nausea and vomiting. Nausea and vomiting were assessed on the first postoperative day, and 120 patients were enrolled. They stated that dexamethasone did not reduce nausea and vomiting [25]. In our study, the combination between dexamethasone and dexmedetomidine reduced postoperative nausea and vomiting. Geng *et al*. studied the effect of dexmedetomidine on 65 adults undergoing laparoscopic surgery with 0.5 mg/kg dexmedetomidine before anesthesia until the end of the surgery, and they found that dexmedetomidine reduced postoperative nausea but had no effect on vomiting 24 hours after surgery [26]. In our study, a combination of dexamethasone and dexmedetomidine reduced postoperative nausea and vomiting. In our study, however, nausea and vomiting were assessed up to 4 hours postoperatively. In 2015, Bakri *et al*. performed a study on 86 patients in Sudan entitled “Comparing dexmedetomidine and dexamethasone in preventing nausea and vomiting after laparoscopic cholecystectomy”. The authors showed that dexmedetomidine reduced the severity and incidence of postoperative nausea and vomiting as the patients in the dexmedetomidine group had less pain in the first 24 hours after surgery [27]. In our study, the combination between dexamethasone and dexmedetomidine reduced postoperative nausea and vomiting. In a meta-analysis from 2015, Liang *et al*. studied the effect of dexmedetomidine on postoperative PONV, using data from Pubmed and Embase databases in a study of 6480 patients, and found that dexmedetomidine had a greater effect on PONV inhibition than placebo, but could not cover all post-anesthetic complications [28]. In our study, the combination of dexamethasone and dexmedetomidine reduced the incidence of postoperative nausea and vomiting. In their study from 2010 performed on 50 patients, Banihashem *et al*. found that dexamethasone and ondansetron reduced the incidence of nausea, vomiting, and itching of intrathecal meperidine in women undergoing elective cesarean section [29].

In our study, the combination of dexamethasone and dexmedetomidine reduced the incidence of postoperative nausea and vomiting. Blood pressure and heart rate values were lower in the dexmedetomidine group at all times and the combination group compared to the dexamethasone group. The combination of dexmedetomidine and dexamethasone reduced postoperative nausea and vomiting in patients. However, further studies with larger sample sizes are recommended, and it is worthy of considering that nausea and vomiting may be recorded starting from 24 hours postoperatively.
